# Dataset on factors affecting social media use among school principals for educational leaderships

**DOI:** 10.1016/j.dib.2021.107654

**Published:** 2021-12-03

**Authors:** Lantip Diat Prasojo, Lia Yuliana, Awanis Akalili

**Affiliations:** Universitas Negeri Yogyakarta, Indonesia

**Keywords:** Social media, Use, School principals, Educational leadership

## Abstract

This dataset was presented to explore the relationships between predictors of an extended theory of acceptance model regarding social media use for educational leadership. Variables, namely subjective norms, supporting conditions, attitudes, perceived ease of use, perceived usefulness, and use of social media, were involved. A survey approach was the approach for the data collection (n. 257). The instrument for the survey was adapted from prior studies, validated through the face and content validity. The examination of loading values, reliability, convergent validity, and discriminant validity was conducted for the measurement model. The dataset of the current study benefits educational stakeholders to issue policy regarding technology use like social media in education, school principals to explore social media use for educational leadership, and future academicians to use the valid and reliable items of the instrument.

## Specifications Table

**Table:** 

Subject	*Social science, education*
Specific subject area	Social Media; School Principals; Educational Leadership
Type of data	Tables Figure
How the data were acquired	Adaptation, Face and content validity, and measurement model;
Data format	Raw Analyzed Filtered
Description of data collection	The instrument involved in this study was developed from the adaptation of prior studies. It was validated through the face and content validity. Through the measurement approach, the instrument was validated and assessed for reliability. The assessment of loading, reliability, convergent validity, and discriminant validity was carried out.
Data source location	Province: Yogyakarta Country: Indonesia Latitude and longitude (and GPS coordinates) for collected samples/data: Latitude: −7.797068 Longitude: 110.370529
Data accessibility	Repository name: Mandeley Data Data identification number: DOI: http://dx.doi.org/10.17632/p36889bm4w.2 Direct URL to the data: http://dx.doi.org/10.17632/p36889bm4w.2

**Value of the Data**

• The dataset of the current study benefits educational stakeholders to issue policy regarding technology use like social media in education,
• School principals can explore social media use for educational leadership, and
• Future academicians might use the valid and reliable items of the instrument for future research.

## 1 Data Description

The data files included in this study are established based some steps of data adaptation and purification, namely adaptation, Face and content validity, and measurement model. In the adaptation process of the instrument, the current dataset included 20 indicators for 6 variables; 4 indicators of perceived usefulness, 3 indicators of perceived ease of use, 4 indicators of subjective norms, 4 indicators of supporting conditions, 3 indicators of attitudes, and 4 indicators of the use of social media. A 5-point Likert scale, 1 (strongly disagree) and 5 (strongly agree), was administered [1]—the instrument from the original scales of TAM [2] and other relevant studies [3,4]. To suit the topic, social media for instructional leadership in the Indonesian context, revisions, and changes for the words were done the respondents' full comprehension. As part of content validity, five experts assessed the indicators for content validity [5,6]. Four principals were also invited to explore their perspectives of the indicators for face validity. Three indicators from perceived usefulness, attitudes, and supporting conditions were respectively eliminated after the discussion sessions.

## 2 Experimental Design, Materials and Methods

**Fig. 1:**
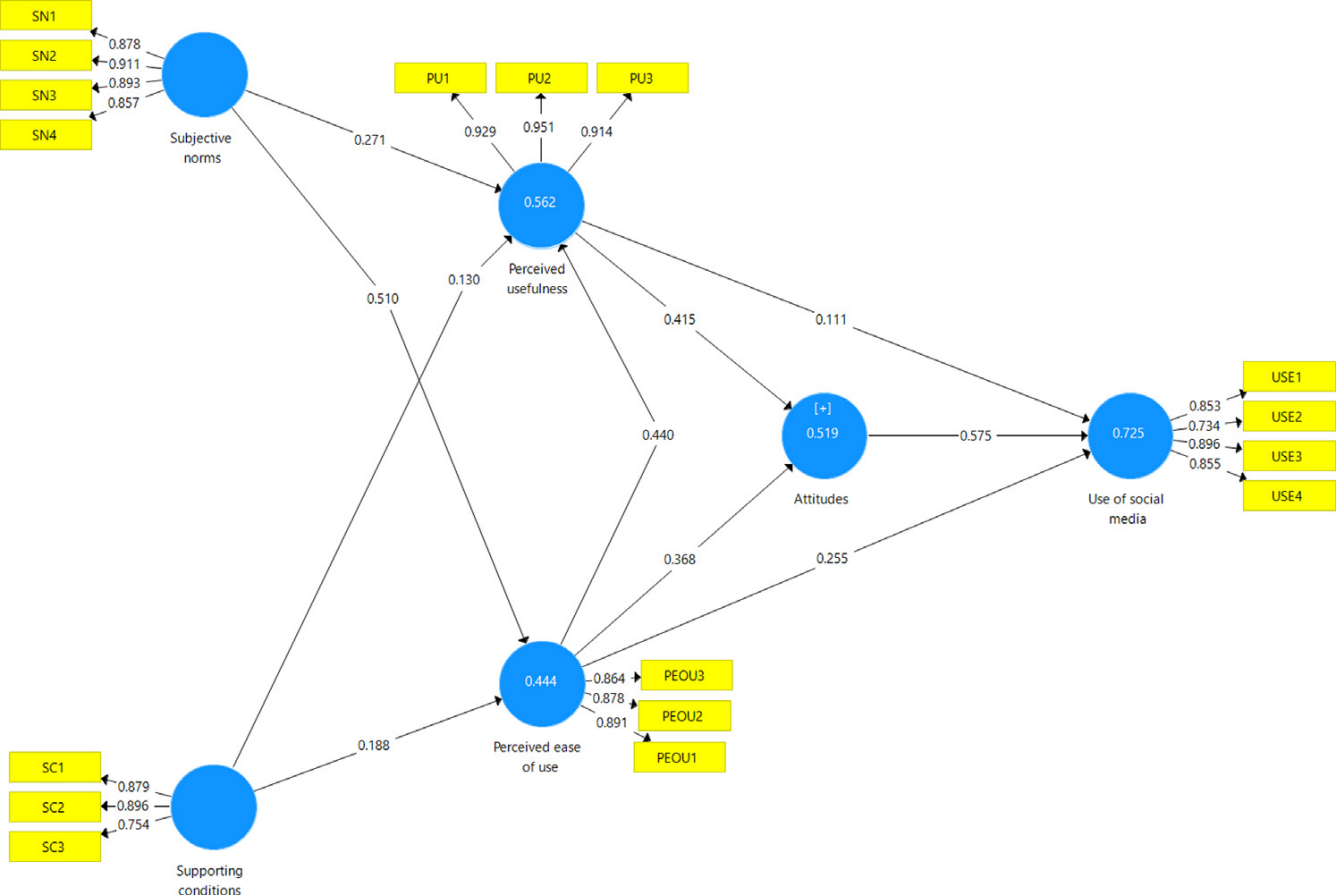
Measurement model of school principals perception on factors affecting social media use for educational leadership

The adaptation and translation of the instrument were made before the initial stage of the validity process [2], [3], [4]. Following the processes, the instrument was discussed with five experts and four users for the face and content validity to suit the context and setting of the dataset [7]. From the data collection process, 257 responses were obtained through simple random sampling; responses were gathered through online survey (Table 1). The assessment of the q-q plot was conducted to assess the normality of the data; data were normal, and no missing data were detected. The primary analysis for the dataset was computed in SmartPLS 3.3 by assessing load values, internal consistency reliability, convergent, and discriminant validity (Tables 2–4). All values are satisfactory for the loading (>.500) and reliability (α, CR, and Rho_A > .700) [8]. The Average Variance Extracted (AVE) values were reported for the convergent validity; values of ≥.500 are evidence of the emergence of the convergent validity. Cross-loading and HTMT are informed to evaluate discriminant validity. All values extend the suggested value. Loads on a construct are required to be greater than their cross-loads (Table 3). HTMT values should be below 0.900 that could be a sign of the discriminant validity (Fig. 1).

**Tabel 1:** Respondents.

Category	Sub-category	n
Gender	Male	169
	Female	108
Age	20–30	26
	31–40	69
	>40	192
Education	Bachelor	134
	Masters	136
School level	Primary school	39
	High school	239

**Table 2:** Construct reliability and validity.

	Cronbach's Alpha	rho_A	Composite Reliability (CR)	Average Variance Extracted (AVE)
Attitudes	0.873	0.875	0.940	0.887
Perceived ease of use	0.852	0.859	0.910	0.771
Perceived usefulness	0.924	0.924	0.945	0.868
Subjective norms	0.907	0.908	0.935	0.783
Supporting conditions	0.799	0.823	0.882	0.715
Use of social media	0.856	0.870	0.903	0.700

**Table 3:** Outer loading and cross-loading.

Items	Attitudes	Perceived ease of use	Perceived usefulness	Subjective norms	Supporting conditions	Use of social media
AT1	*0.945*	0.629	0.667	0.726	0.656	0.780
AT2	*0.939*	0.606	0.593	0.686	0.660	0.758
PEOU1	0.623	*0.891*	0.713	0.610	0.538	0.686
PEOU2	0.534	*0.878*	0.531	0.552	0.486	0.575
PEOU3	0.562	*0.864*	0.565	0.558	0.507	0.596
PU1	0.627	0.610	*0.929*	0.617	0.552	0.629
PU2	0.630	0.660	*0.951*	0.636	0.583	0.631
PU3	0.616	0.668	*0.914*	0.592	0.532	0.621
SC1	0.590	0.518	0.545	0.670	*0.879*	0.616
SC2	0.675	0.560	0.534	0.729	*0.896*	0.645
SC3	0.492	0.381	0.424	0.550	*0.754*	0.503
SN1	0.662	0.575	0.577	*0.878*	0.693	0.655
SN2	0.696	0.588	0.604	*0.911*	0.708	0.681
SN3	0.661	0.593	0.596	*0.893*	0.701	0.652
SN4	0.636	0.563	0.560	*0.857*	0.636	0.646
USE1	0.716	0.689	0.643	0.664	0.603	*0.853*
USE2	0.556	0.500	0.440	0.493	0.460	*0.734*
USE3	0.775	0.623	0.588	0.640	0.626	*0.896*
USE4	0.663	0.542	0.560	0.680	0.639	*0.855*

**Table 4:** HTMT.

	Attitudes	Perceived ease of use	Perceived usefulness	Subjective norms	Supporting conditions
Attitudes					
Perceived ease of use	0.756				
Perceived usefulness	0.745	0.774			
Subjective norms	0.842	0.743	0.721		
Supporting conditions	0.830	0.695	0.690	0.899	
Use of social media	0.893	0.820	0.751	0.841	0.838

## Ethics Statement

Informed consent was distributed and collected during data collection.

## CRediT authorship contribution statement

**Lantip Diat Prasojo:** Conceptualization, Methodology, Software, Data curation, Investigation. **Lia Yuliana:** Conceptualization, Supervision. **Awanis Akalili:** Data curation, Writing – original draft.

## Declaration of Competing Interest

The authors declare that they have no known competing financial interests or personal relationships which have or could be perceived to have influenced the work reported in this article.
